# The network of DAB2IP-miR-138 in regulating drug resistance of renal cell carcinoma associated with stem-like phenotypes

**DOI:** 10.18632/oncotarget.17756

**Published:** 2017-05-09

**Authors:** Eun-Jin Yun, Jiancheng Zhou, Chun-Jung Lin, Shan Xu, John Santoyo, Elizabeth Hernandez, Chih-Ho Lai, Ho Lin, Dalin He, Jer-Tsong Hsieh

**Affiliations:** ^1^ Department of Urology, University of Texas Southwestern Medical Center, Dallas, TX 75390, USA; ^2^ Department of Urology, Shaanxi Provincial People's Hospital, Xi’an, Shaanxi 710068, China; ^3^ Department of Urology, The First Affiliated Hospital, Medical School of Xi'an Jiaotong University, Xi'an 710049, China; ^4^ Department of Microbiology and Immunology, Chang Gung University, Taoyuan 333, Taiwan; ^5^ Department of Life Sciences, National Chung Hsing University, Taichung 402, Taiwan; ^6^ Graduate Institute of Cancer Biology, China Medical University Hospital, Taichung 40447, Taiwan, Republic of China

**Keywords:** miR-138, DAB2IP, cancer stem cell, drug resistance

## Abstract

Targeted therapy is a standard of care for metastatic renal cell carcinoma (RCC) but the response rate is not overwhelmed, which only prolongs a short survival of patients due to the onset of therapeutic resistance. Although the mechanisms are not fully understood, the presence of cancer initiating cells (CIC) may underlie the drug resistance. Nevertheless, identifying CIC phenotypes with its biomarkers in RCC appear to be diverse and controversial from many reports. In this study, we took a different approach to focus on the regulatory mechanism in RCC-CIC and unveil DAB2IP-mediated miR-138 expression that plays a critical role in modulating stem-like phenotypes in RCC via targeting the ABC transporter (ABCA13) as well as oncogenic histone methyltransferase EZH2 while down regulation of miR-138 gene expression in RCC is due to epigenetic gene silencing by DNA methyltransferase 1 (DNMT1). We also characterize the individual mechanism by which ABCA13 in RCC-CIC contributes to its drug resistance and. EZH2 maintain stem-like phenotypes. Noticeably, elevated expression of ABCA13 and EZH2 is correlated with overall survival of RCC patients, which can be used as potential prognostic markers. Taken together, this study demonstrates a potent and unique pathway of DAB2IP-mediated miR-138 in modulating CIC phenotypes during RCC progression and also offers a new therapeutic strategy of targeting drug resistant RCC.

## INTRODUCTION

The incidence of renal cell carcinoma (RCC) has been steadily rising over the past years [1]. RCC is by far the most lethal urologic malignancy because it's metastatic disease due to the resistance to chemotherapy, targeted therapy and radiation, and is largely incurable with a 5-year survival rate of less than 10%. In spite of recent advancements and clear clinical benefits observed with first-line targeted agents in RCC in terms of improved survival, a subset of patients do not appear to experience clinical benefit from targeted therapy and eventually acquired drug resistance. The mechanism of RCC therapeutic resistance is not fully understood, though it is speculated that the ATP-binding cassette (ABC)/p-glycoprotein transporter system in RCC may play a role [2–4]. Recently, cancer stem cell (CSC) model provides additional mechanism of drug resistance. CSC is considered as a cancer initiating cell (CIC) with poorly differentiated, immortal, self-renewal capabilities that can give rise to numerous progeny cells [5] and resist to conventional therapeutics. It has been observed in the end stage of many solid tumors including prostate cancer [6], breast cancer [7], ovarian cancer [8], and brain tumors [9]. In RCC, the role of CSC/CIC is still debatable because of lacking defined surface markers and clear pathway(s) to identify this population [10]. Because CSC/CIC is believed to play a crucial role in tumor recurrence after conventional cancer therapy due to their immortality and the resistant to chemo- or radio-therapy, it is critical to unveil the regulation mechanisms of CSCs to develop effective therapeutic strategies that are more specifically directed against CSCs.

In this study, we newly demonstrated that the loss of DAB2IP in RCC increased stem-like phenotypes such as cancer initiating ability and drug resistance. Previously, we identified DAB2IP is a novel tumor suppressor commonly lost in every subtype of RCC [11]. Here, we further identified DAB2IP-mediated microRNA-138 (miR-138) pathway to target EZH2 and ABCA13 expression that underlined the stem-like phenotypes associated with drug resistance of RCC cells. Clinical evidence showed that miR-138 expression significantly reduced in RCC specimens. Also, overall survival of RCC patients was positively correlated with both EZH2 and ABCA13 expression. A unique function of DAB2IP protein is identified through its direct interaction with epigenetic enzymes such as DNA methyltransferase 1 (DNMT1) and histone deacetylase (HDAC2), which can prevent both enzymes from binding to the miR-138 gene promoter leading to the expression of miR-138. In conclusion, both ABCA13 and EZH2 are key players in eliciting stem-like phenotypes of RCC and potential prognostic markers, which may also provide a new therapeutic strategy against RCC drug resistance.

## RESULTS

### Down regulation of miR-138 promotes stem-like phenotypes in RCC

**Figure 1:**
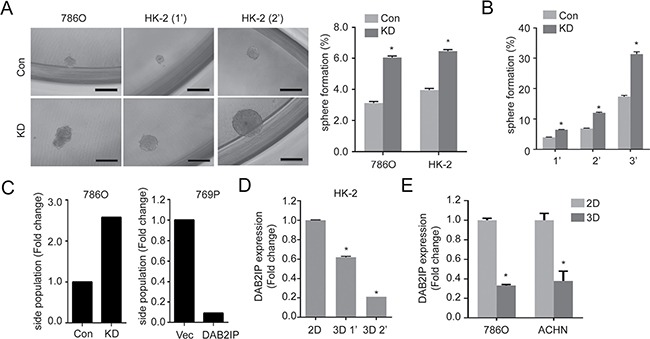
Loss of DAB2IP increases stem-like phenotypes in RCC (**A**) Cells were cultured with sphere forming condition in ultra-low attachment plate for 14 days. Spheres were photographed, and the numbers of spheres were counted, and sphere forming ability was calculated based on the original seeding number. Con, control cells; KD, DAB2IP knock-down cells; 1’, the first generation spheres; 2′, the second generation spheres. The asterisk (*) indicates statistical significance (*p* < 0.01) between KD and Con. (**B**) Sphere forming ability was determined after serial passages. (**C**) Cells were stained with Hoechst 33342 and side population (SP) was determined using flow cytometry. (**D** and **E**) DAB2IP expressions were compared in HK-2 cells (D), 786O or ACHN RCC cells (E) cultured in monolayer (2D) or sphere condition (3D).

In RCC, the presence or role of CSC/CIC remained undefined. Previously, we demonstrated the comprehensive inhibitory effect of DAB2IP on various CSC subpopulation in prostate cancer via different mechanism of action [12]. In this study, similar to the results from prostate cancer, we also observed that DAB2IP-deficient renal cells acquired CSC potential based on CSC hallmarks [10] such as sphere-forming ability under ultralow-attachment growth condition (Figure 1A and 1B) and increased side population (SP) (Figure 1C). In addition, we placed the renal cell lines such as HK-2, 786O and ACHN under ultralow-attachment growth condition to enrich their sphere forming abilities by passaging these cells continuously. Noticeably, a significant decreased DAB2IP expression was detected in 3D renal sphere cells compared with 2D monolayer culture (Figure 1D and 1E), suggesting that DAB2IP plays an inhibitory role in renal sphere cell formation. We therefore screened potential surface biomarker based on reported CSC markers. Using these renal spheres exhibiting stem-like phenotypes, we have screened CSC markers such as CD44, CD117 or CD133 (Supplementary Figure 1A) and no consistent results were obtained, which prompted us to use different strategies to search potential biomarker(s).

**Figure 2:**
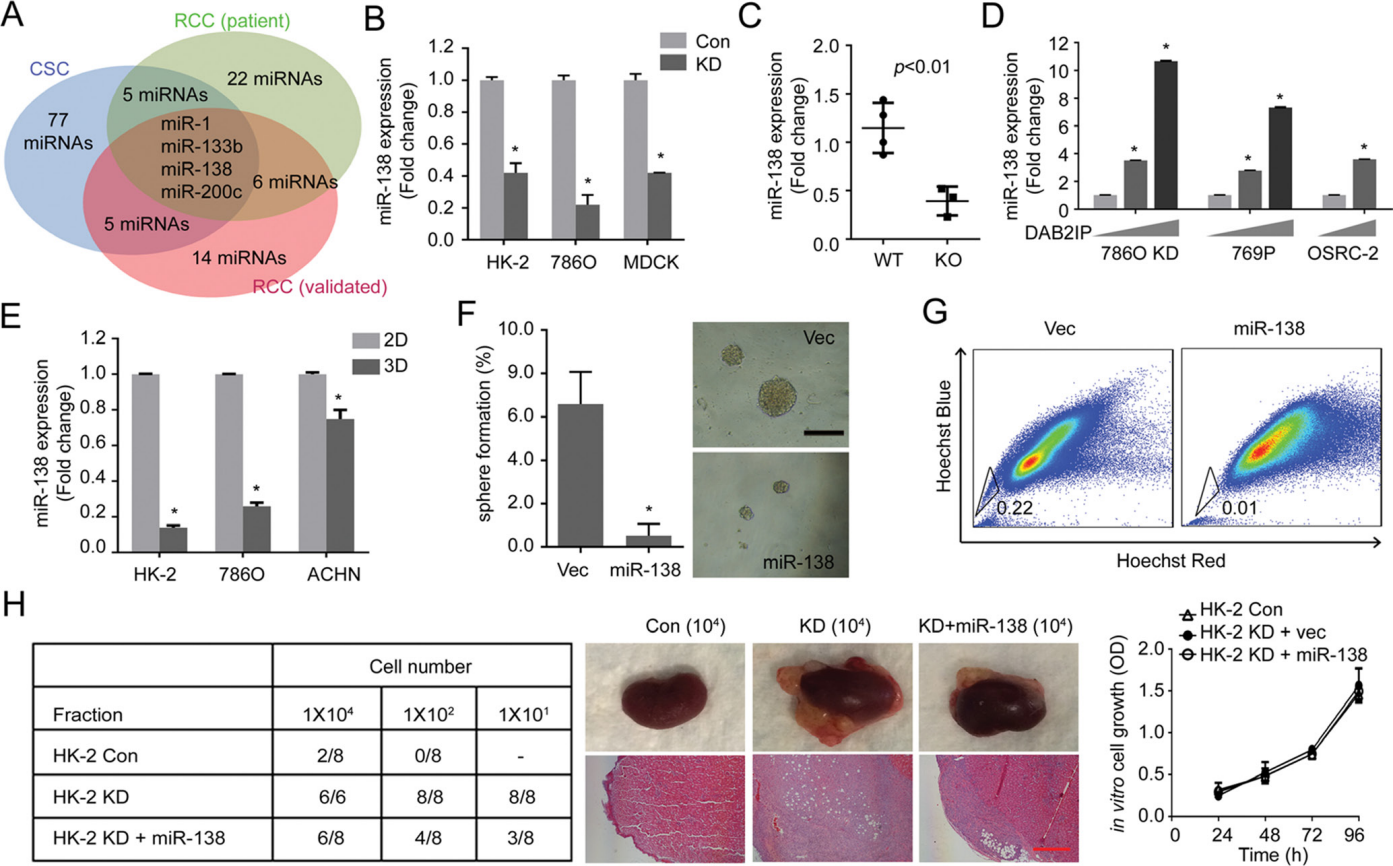
miR-138 is regulated by DAB2IP to suppress stem-like phenotypes in RCC (**A**) Potential miRNAs candidates were analyzed from different data sources: DAB2IP-regulated miRNAs (Blue), Screening of tumor suppressive miRNAs from RCC patients (Green) [39], and Profiling of miRNAs during RCC progression (Red) [40]. (**B** and **C**) Expression levels of miR-138 were determined in Con vs. KD renal cells (B), and in wild type (WT) vs. DAB2IP knock-out (KO) mice tissues (C). (**D**) Effect of DAB2IP on the expression levels of miR-138 expression. (**E**) miR-138 expressions were compared between monolayer (2D) and sphere (3D) culture condition of HK-2, 786O or ACHN cells. (**F** and **G**) HK-2 KD cells were transfected with miR-138 expression vector. The sphere forming ability (E), and SP (G) were determined. (**H**) Cancer-initiating ability of each subline was determined using orthotopic xenograft model after 8 weeks post-injection. Scale bar, 250 μm.

MicroRNAs (miRNAs) is known to target specific gene expression post-transcriptionally and has been shown to be involved in CSCs [13, 14]. To assess a possible regulatory mechanism, we screened DAB2IP-regulated miRNAs using DAB2IP modulated RCC cell lines (Figure 2A, Blue). Then, the results were compared with two publically reported miRNA profiles using clinical specimens to narrow down potential candidates (Figure 2A, Green and Red). Among these candidates, miR-138 expression was further validated from *in vitro* cell models (Figure 2B) and DAB2IP knock-out (KO) mice model (Figure 2C). As expected, the presence of DAB2IP is able to induce miR-138 expression in 786O KD, 769P and OSRC-2 cells (Figure 2D), indicating that miR-138 is a downstream gene induced by DAB2IP. In spheres derived from these RCC lines, miR-138 expression were significantly suppressed (Figure 2E). Furthermore, 293T, a tumorigenic human embryonic kidney cells, has been known to form spheres with enriched CSC markers [15]; both DAB2IP and miR-138 expression were significantly reduced in 3D renal spheres compared with 2D monolayer cells (Supplementary Figure 1B).

We further determined the effect of miR-138 on stem-like properties of RCC cells. Ectopic expression of miR-138 or DAB2IP in 293T cells dramatically decreased their sphere-forming abilities (Supplementary Figure 1C). Moreover, elevated expression of miR-138 suppresses sphere formation as well as SP of HK2-KD cells (Figure 2F and 2G). In contrast, knocking down endogenous miR-138 in 786O and HK-2 cells using an antagomiR system resulted in an enhanced sphere-forming ability (Supplementary Figure 1D). In *vivo*, DAB2IP KD cells exhibited CIC property; this cell remained highly tumorigenic at very low cell numbers using an orthotopic model (Figure 2H). Elevated miR-138 in KD cell significantly decreased its CIC ability *in vivo* but didn't alter its *in vitro* growth rate (Figure 2H), suggesting that miR-138 is a potent suppressor of CIC potential rather than growth inhibition.

### DAB2IP epigenetically regulates miR-138 expression in RCC

**Figure 3:**
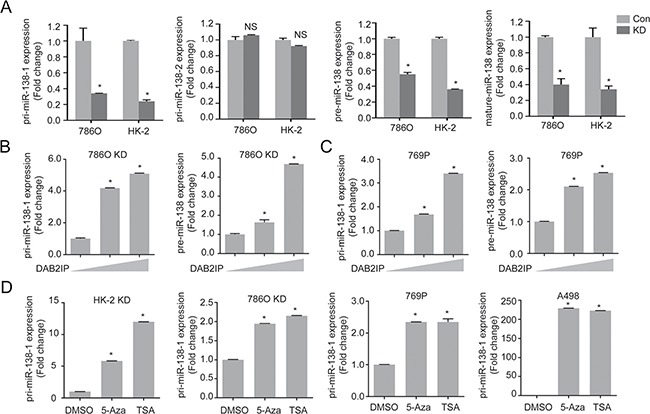
DAB2IP epigenetically regulates miR-138 expression (**A**) Expression levels of primary, precursor, and mature miR-138 were compared in 786O and HK-2 sublines (Con, KD). (**B** and **C**) 786O KD (B) and 769P (C) cells were transfected with increasing dosage of DAB2IP for 48 h, and relative expressions of primary and precursor miR-138 were analyzed by qRT-PCR. (**D**) Cells were treated with 5-Aza (5 μM) and TSA (100 nM) for 72 h, and pri-miR138-1 expressions were analyzed.

It is known that human miR-138 derives from two separate genomic locations: chromosome 3 and 16 as miR-138-1 and miR-138-2, respectively. As shown in Figure 3A, the results revealed that the primary transcript of miR-138-1 gene (pri-miR-138-1) was significantly decreased in KD cells (∼70%), yet there were no significant changes for pri-miR-138-2, suggesting that decreased precursor and mature miR-138 levels in DAB2IP KD cells is likely a result of decreased pri-miR-138-1 expression. Indeed, an incremental expression of DAB2IP in 786O KD and 769P cells increased pri-miR-138-1 expression at both precursor and mature miR-138-1 levels (Figure 3B and 3C). These data suggest that DAB2IP is an inducer of pri-miR-138-1 gene expression.

### DNMT1 suppresses miR-138 expression by direct binding to promoter region

**Figure 4:**
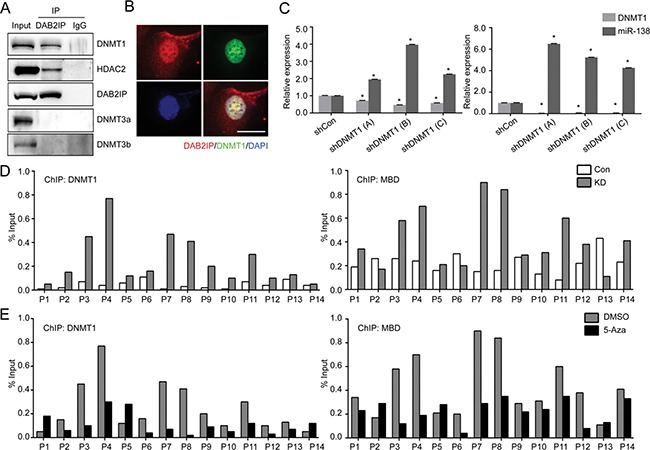
DNMT1 suppresses miR-138 expression by direct binding to the promoter region (**A**) Nuclear proteins were extracted from wild type 786O cells and interaction of DAB2IP with DNMTs or HDAC2 were validated by IP. (**B**) Co-localization of DAB2IP and DNMT1 in the nuclei of 786O cell lines was determined by immunofluorescent staining. Scale bar = 25 μm. (**C**) DNMT1 was knock-downed in 786O KD and 769P cells, and miR-138 expression was measured by qRT-PCR. (**D**) The status of DNMT1 (left panel) or MBD (right panel) binding to the miR-138 gene promoter region in 786O Con and KD were evaluated by ChIP assay. (**E**) 786O KD cells were treated with 5-Aza (5 μM) for 72 h, and the effect of 5-Aza treatment on DNMT1 or MBD binding to the miR-138 promoter region were determined by ChIP assay.

To further investigate the regulatory mechanism of miR-138 gene expression by DAB2IP, we observed that the expression levels of pri-miR-138-1 in DAB2IP KD cells could be recovered by DNA hypomethylation agent (5-Aza-2′-deoxycytidine; 5-Aza) or histone deacetylase inhibitor (Trichostatin; TSA) (Figure 3D), implying a possible inhibitory effect of DAB2IP on the expression of epigenetic enzymes. However, we further ruled out the possibility that DAB2IP can suppress the expression of several epigenetic enzymes using cDNA array and Western blot analyses (Supplementary Figure 2). We then hypothesized that DAB2IP might interact with these enzymes to prevent their binding to the miR-138-1 gene promoter. By performing immuneprecipitation (IP) and mass-spectrometry (MS) analyses, results showed several epigenetic enzymes, including DNMT1 and HDAC2, could interact with DAB2IP (Supplementary Table 1). We further validated these results using IP and immunofluorescent (IMF) staining to show the interaction between DAB2IP and DNMT1 or HDAC2 but not DNMT3 (Figure 4A and 4B). DNMT1 is, the primary enzyme responsible for maintaining CpG methylation leading to gene inactivation, significantly elevated in several types of cancer including RCC [16–18]. To explore whether the interaction between DAB2IP and DNMT1 could impact on miR-138 expression, DNMT1 expression was knock-downed using several shRNA constructs. Indeed, loss of DNMT1 significantly increased miR-138 expression in both 786O KD and 769P cells (DAB2IP^−^), indicating DNMT1 plays a critical role in miR-138 regulation (Figure 4C). In addition, ChIP data clearly demonstrated direct binding of DNMT1 to several miR-138-1 promoter regions was significantly increased in the absence of DAB2IP (Figure 4D left panel); these regions correspond with methyl-CpG binding protein (MBD) binding regions (Figure 4D right panel). In contrast, the presence of DAB2IP had the opposite effect (Supplementary Figure 3A). For HDAC2, the overall binding to the miR-138-1 promoter was elevated in DAB2IP-KD cells (Supplementary Figure 3B). Moreover, the treatment of 5-Aza in 786O KD cells decreased binding of DNMT1 and MBD to miR-138-1 promoter regions (Figure 4E). Together, DAB2IP has a new function as a critical epigenetic modulator for miR-138 expression by sequestering DNMT1 and HDAC2 from their bindings to the promoter region.

### miR-138 suppresses ABCA13 and EZH2 by direct targeting of 3′UTR

**Figure 5:**
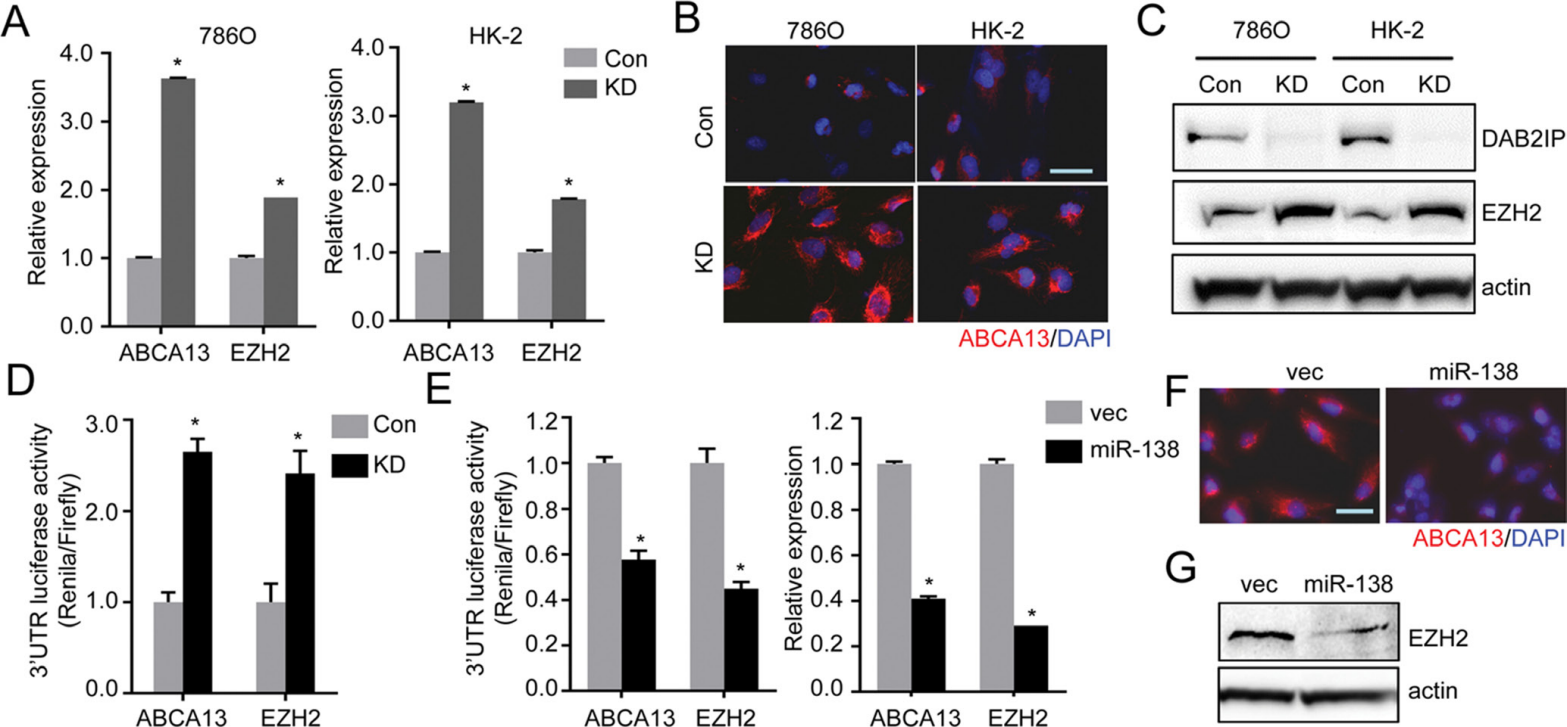
miR-138 targets ABCA13 and EZH2 mRNA via the 3′-UTR regions (**A**–**C**) Expression levels of ABCA13 and EZH2 in RCC cells were analyzed by qRT-PCR (A), or immunofluorescence staining (B), or Western blot analysis (C). (**D**) Luciferase reporter activities in 786O cells transfected with either ABCA13 or EZH2 3′UTR reporter construct for 48 h. The luciferase reporter activity was measured with the dual luciferase assay. (**E**) The inhibitory effect of miR-138 on 3′UTR reporter activities or mRNA expression of ABCA13 and EZH2 in HK-2 KD cells were analyzed. (**F** and **G**) The protein expressions of ABCA13 (F) and EZH2 (G) were determined in HK-2 KD transfected with miR-138 expression vector.

To explore the role of miR-138 in suppressing CSC/CIC phenotype, we screened targets of miR-138 using TargetScan, PicTar and Miranda and identified two candidates: ABC transporter ABCA13 and oncogenic histone methyltransferase EZH2 expression commonly elevated in DAB2IP KD cells (Figure 5A–5C). We further demonstrated that the activities of ABCA13 and EZH2 3′-untranslated region (3′-UTR) reporters were significantly higher in KD cells than in Con cells and the presence of miR-138 could suppress the activities of 3′-UTR reporters of these genes (Figure 5D and 5E), which also corresponded to the protein expression of ABCA13 or EZH2 (Figure 5F and 5G) respectively. Similar results from a variety of RCC cell lines support the suppressive effect of DAB2IP or miR-138 on ABCA13 and EZH2 mRNA expression and 3′-UTR reporters’ activity (Supplementary Figure 4).

### EZH2 and ABCA13 are critical for maintaining CSC/CIC phenotypes of RCC and associated with overall survival of RCC patients

**Figure 6:**
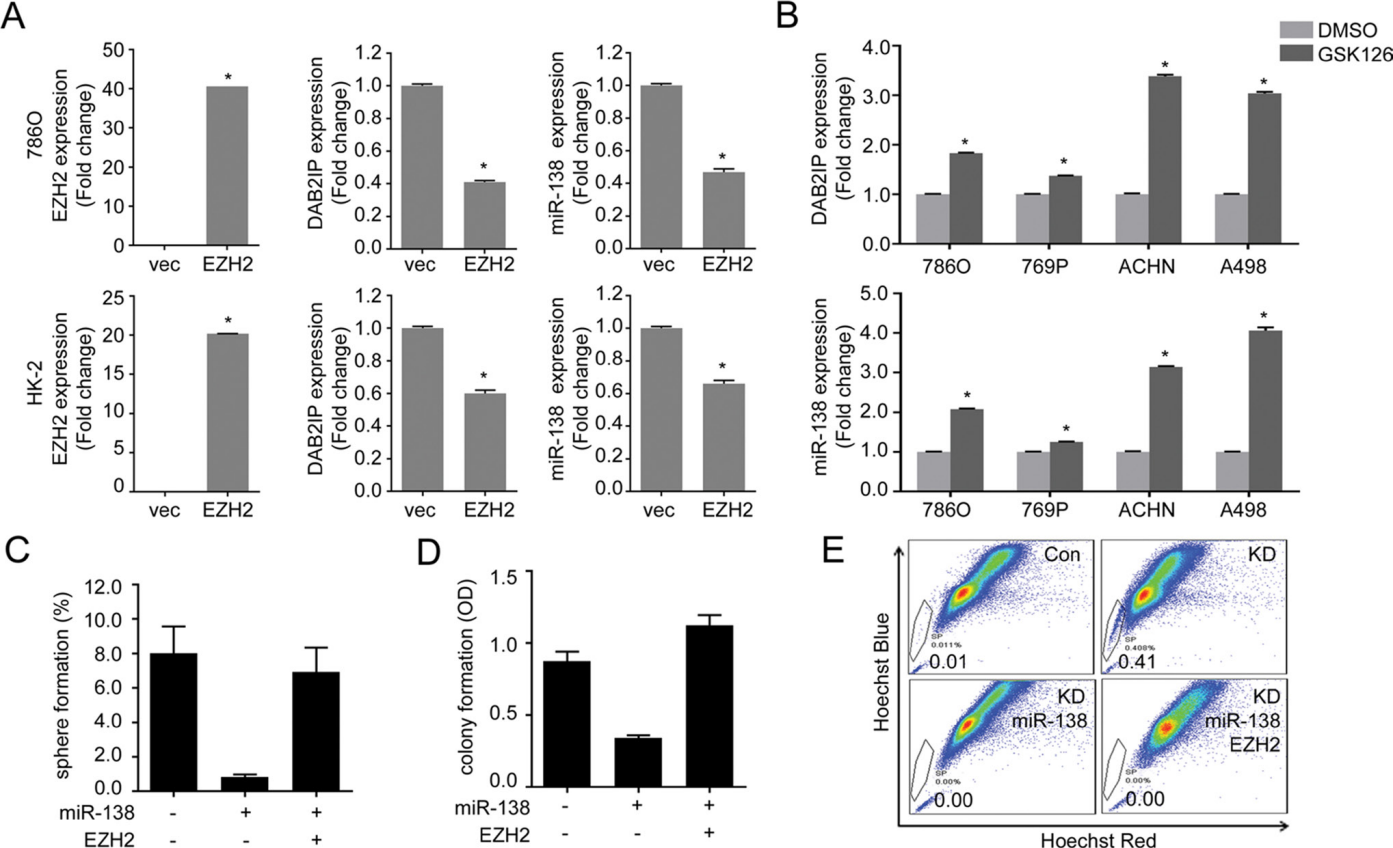
EZH2 plays a critical role in maintaining stem-cell like properties of renal cells (**A**) Cells were transfected with EZH2 expression plasmid for 48 h then expression of DAB2IP and miR-138 levels were determined by qRT-PCR. (**B**) Cells were treated with EZH2 inhibitor GSK126 (1 μM) for 48 h, then DAB2IP and miR-138 expressions were determined. (**C**–**E**) Cells were transfected with miR-138 and/or EZH2 expression vector as indicated, the sphere forming ability (C), colony forming ability (D), or SP (E) was determined separately.

Knowing EZH2 and ABCA13 are the target genes for miR-138, we investigated the role of EZH2 and ABCA13 in CSC/CIC phenotypes. Previous studies have shown that EZH2 is a master regulator for cancer stemness [19, 20], and that EZH2 expression is associated with increased metastatic potential, as well as poor clinical outcome in RCC patients [21]. We previously reported that EZH2 could inhibit DAB2IP gene expression by direct interaction of EZH2 with the DAB2IP promoter region [22]. We found EZH2 significantly reduced both DAB2IP and miR-138 expression (Figure 6A), whereas the treatment with an EZH2 inhibitor (GSK126) induced their expression (Figure 6B). It appears likely this reciprocal regulation between EZH2 and mir-138 is critical for stem-like phenotypes in RCC. For example, the presence of miR-138 significantly decreased the number of sphere, colony formation and SP (Figure 6C–6E). Interestingly, overexpression of miR-resistant EZH2 restored both sphere and colony formation (Figure 6C and 6D) but not SP (Figure 6E).

**Figure 7:**
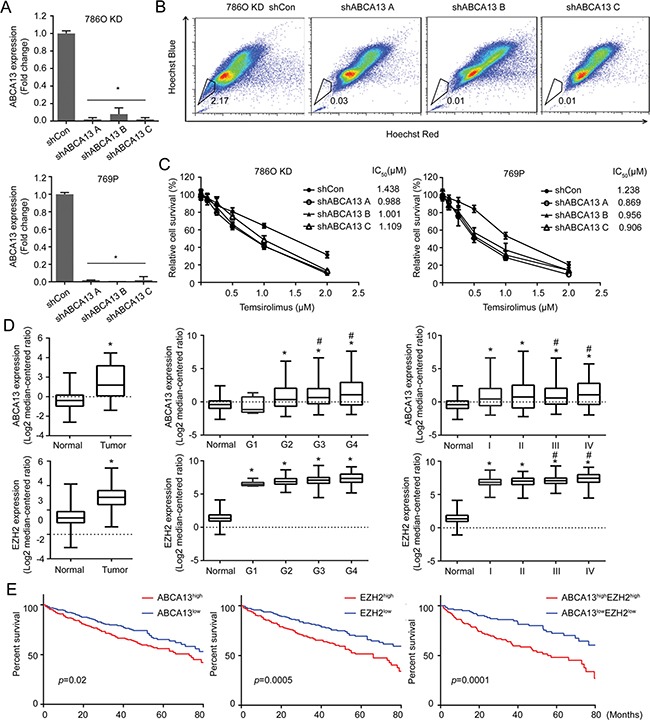
ABCA13 confers drug resistance of RCC and correlates with the overall survival of RCC patients (**A**) ABCA13 was knock-downed in 786O KD and 769P cells, and knock down efficiency was measured by qRT-PCR. (**B**) SPs were determined and compared in 786O KD cells transfected with shCon and shABCA13 plasmid. (**C**) 786O KD or 769P cells were transfected with shCon or shABCA13 plasmid then treated with Temsirolimus for 72 h and the relative cell survival was measured by MTT assay for determining IC_50_ using Graphpad Prism. (**D**) The levels of miR-138, ABCA13 and EZH2 expression in normal versus tumor tissues from TCGA were compared. The asterisk (*) indicates statistical significance (*p* < 0.05) between tumor and normal; # indicates statistical significance (*p* < 0.05) between higher grade and G1. (**E**) Overall survival of high- and low- ABCA13/EZH2 groups from TCGA was analyzed. *N* = 468, *p* value was obtained from two-sided log-rank tests.

SP is considered a characteristic of stemness due to the presence of ABC transporters that can efflux drug from stem cells to gain the resistance [23]. Therefore, we knocked down ABCA13 (Figure 7A) to determine its effect on drug efflux. As expected, ABCA13 KD cells dramatically decreased drug efflux (Figure 7B). ABCA13 KD cells also became more sensitive to chemotherapeutic drugs such as Temsirolimus and Docetaxel with reduced IC_50_ (Figure 7C; Supplementary Figure 5). In contrast, knocking down endogenous miR-138 in RCC cells induced their resistance to Temsirolimus (Supplementary Figure 5). These results suggest a role for ABCA13 in drug resistance of stem-like RCC. Overall, our study reveals a unique regulatory network between a tumor suppressor protein (DAB2IP) and miRNA (miR-138) that are impenitent in preventing the onset of CSC/CIC phenotypes in RCC.

We further determined any clinical correlation between miR-138 expression and ABCA13 and EZH2 using Cancer Genome Atlas (TCGA) database. Data indicated miR-138 levels were significantly lower in RCC specimens compared to normal kidney tissue, whereas ABCA13 and EZH2 expression were elevated in RCC tissues (Figure 7D; Supplementary Figure 6; *p* < 0.001). Particularly, elevated ABCA13 and EHZ2 expressions were positively correlated with tumor grade and disease progression of RCC (Figure 7D). We found that ABCA13 or EZH2 expression level inversely correlated with the overall survival of RCC patients. Nevertheless, a combination of both ABCA13 and EZH2 markers appeared to have better predictor power (Figure 7E).

## DISCUSSION

CSC known as CIC possess genetic instabilities and embryonic self-renewal capabilities, which could render the progeny cells to evade the killing by conventional therapeutics mainly targeting mitotic cells [3, 5]. Also, an elevated ABC transporter system in CSC expression often attributes to its drug resistance [5]. Although CSC/CIC behaves like an embryonic cell, the mechanisms leading to CSC/CIC appear to be specific to each cancer type. In RCC, some studies have reported the isolation of CSC/CIC [24, 25]; however, the biomarker(s) associated with CSC/CIC, the molecular mechanisms leading to CSC/CIC, and its role in renal carcinogenesis remains controversial. In this study, we unveiled the role and mechanism of DAB2IP, a tumor suppressor commonly lost in each subtype of RCC, in CSC/CIC phenotypes as well as identified the new biomarker with new therapeutic target(s) in RCC.

Furthermore, accumulating lines of evidence suggest that miRNAs regulate tumorigenesis via modulation of CSC/CIC properties and are deregulated in many types of human cancer [26]. Oncogenic miRNAs, such as Lin-28B, miR-9, miR-181, and miR-215, are responsible for tumor initiation or drug resistance by enhancing CSC/CIC properties, while miR-34a, let-7 and miR-200 considered as tumor suppressor that is frequently lost in cancer and CSC/CIC [27, 28]. Our study demonstrates that miR-138 expression was significantly suppressed in sphere cells compared to adherent cells; Many studies have suggested that the CSCs can be enriched within the spheres when cultured in a suspension condition [6, 7, 9, 23]. In addition, down-regulation of miR-138 significantly enhanced CSC/CIC phenotypes in RCC, such as sphere formation, drug efflux and *in vivo* tumorigenic potential. Our results conclude that miR-138 is a key regulator of CSC/CIC in RCC.

The biogenesis and function of miRNAs have been studied extensively [29], however, gene regulation of most miRNAs remains unclear. For miR-138, it appears that epigenetic machinery such as DNMT1 and HDAC is responsible for miR-138 gene slickening in RCC. DNMT1, often forming a complex with HDAC and PCNA, has been implicated in tumorigenic process that may act to silence tumor suppressor genes [16–18]. Epigenetic mechanisms are important regulators of tissue-specific miRNAs expression. For example, both DNA methylation and histone modification are critical in regulation of miRNAs expression during RCC development and metastatic recurrence [30]. Noticeably, we found a specific functional role of DAB2IP engaging in miR-138-1 but not miR-138-2 gene expression by preventing epigenetic complex from binding to miR-138-1 gene promoter, which unveils a new function of DAB2IP in epigenetic modulation of RCC cells.

Several studies have defined the targets of miR-138 such as VIM, RhoC, Hif-1α, CCND1 and Sirt1 [31–33] in cancer types other than RCC, which are often involved in cell migration, EMT, DNA damage repair, senescence. Here, we identified two additional targets; EZH2 and ABCA13 from RCC cells. EZH2 is critical for maintaining CSC phenotypes in RCC, as well as in other cancer types [19, 34]. Several ABC transporters including ABCG2, ABCB1, and ABCC1 have also been associated with drug efflux [35, 36]. A recent study showed an association between higher ABCA13 levels and primary drug resistance, resulting in poor overall survival in ovarian cancer patients [37]. Overexpression of the ABC transporter system has been observed in malignant renal cells derived from the proximal renal tubule [2]. From TCGA data, ABCA13 was associated with the overall survival rates for RCC patients. Thus, we believe that ABCA13 can be a new biomarker for CSC/CIC in RCC.

**Figure 8:**
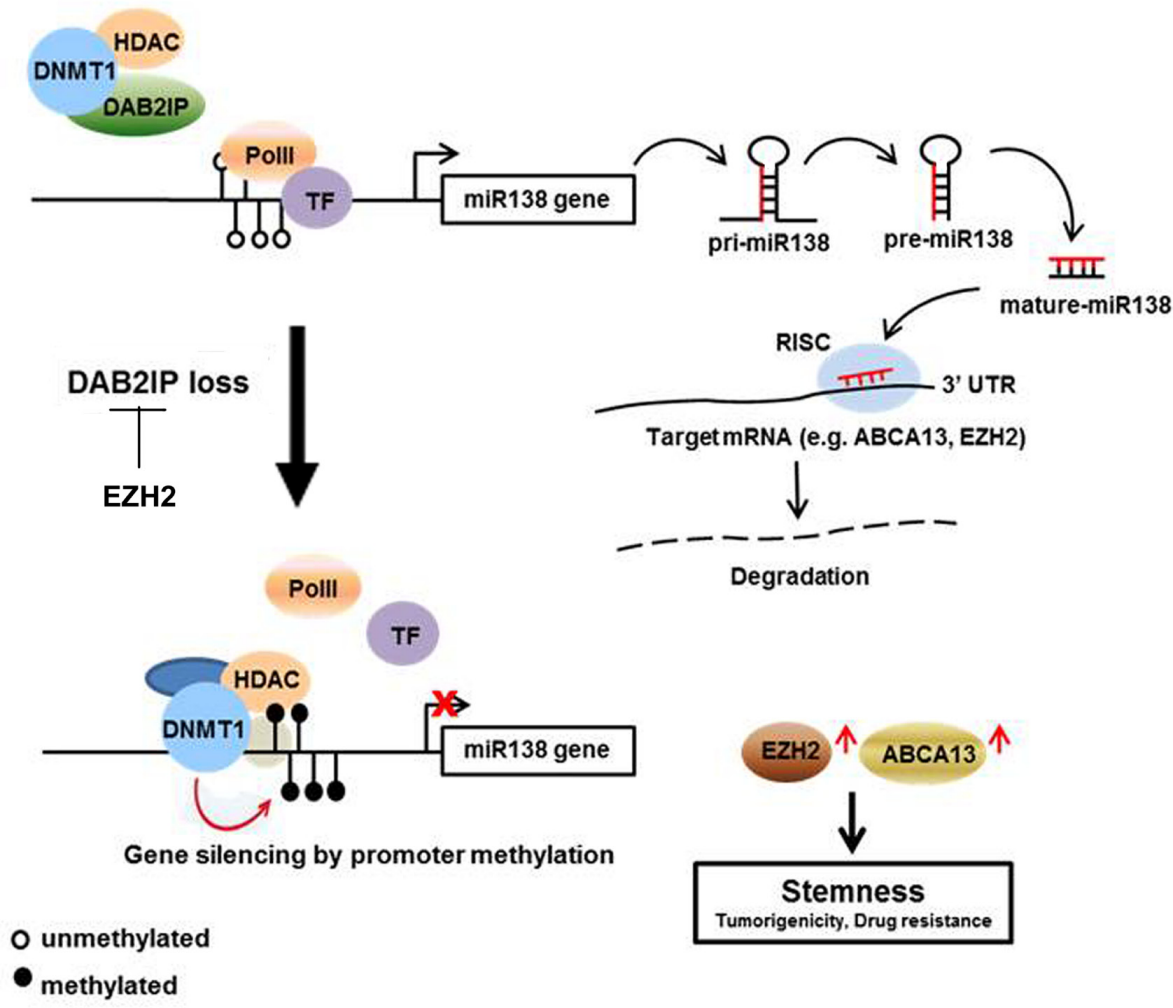
Summary of the regulation mechanism of miR-138 on suppressing RCC-CSCs DAB2IP blocks the binding of DNMT1 and HDAC2 to miR-138 promoter region. This blocking inhibits the methylation of miR-138 promoter, and increased miR-138 expression leads the degradation of ABCA13 and EZH2 which are involved in CSC development. In advanced RCC, loss of DAB2IP allow to the binding of DNMT1 to miR-138 promoter and suppress miR-138.

Our study provides a new mechanistic insight of DAB2IP-mediated miR-138 gene regulation that is critical for the onset of CSC phenotypes associated with the expression of EZH2 and ABCA13 (Figure 8). The expressions of EZH2 and ABCA13 are highly clinical relevant and should be further evaluated as prognostic makers for RCC management. This new regulation mechanism and specific markers associated with chemo-resistance and CSC progression of RCC would be critical for the early detection. Also, new therapeutic strategies based on miR-138, EZH2, and ABCA13 can be used for targeting CSC/CIC in RCC. Selectively targeting CSC could be more effective, and cause fewer side effects, and it would be possible to treat patients with aggressive, resistant, non-resectable tumors as well as preventing the tumor from metastasizing.

## MATERIALS AND METHODS

### Cell culture, plasmid and reagents

Human RCC cell lines 786O, 769P and AHCN cells were cultured in RPMI-1640 (Life Technologies) containing 10% fetal bovine serum (FBS). OSRC-2 cells were maintained in T-media containing 10% FBS at 37^°^C in a 5% CO_2_ atmosphere. HK-2 cells line, human papillomavirus 16 (HPV-16) transformed, non-tumorigenic, human kidney cell line derived from the proximal tubule, was cultured in RPMP-1640 containing 10% FBS. HEK293T cell line, a transformed human embryonic kidney cell, was cultured in Dulbecco's modified Eagle's medium (DMEM, Life Technologies) supplemented with 5% FBS. Cell lines were authenticated using AmpFLSTR^®^Identifier^®^ PCR Amplification kit (Applied Biosystems, Grand Island, NY, USA) and MycoAlert^®^ kit (Lonza Walkersville, Inc. Walkersville, MD, USA) to confirm mycoplasma-free condition. Human miR-138 expression vector and antagomiR were purchased from Origene and GeneCopoeia, respectively. Cells were plated with 70% confluence and transfection was carried out using Xfect (Clontech) according to the manufacture instructions. Hoechst33342 was purchased from Life Technologies, Verapamil from Sigma, and GSK126 from Xcess Biosciences Inc.

### Quantification of miRNA expression

Small RNA was isolated and enriched using the *mirVana* miRNA isolation kit (Ambion) following the manufacturer's protocol, and 200 ng of small RNA was reverse transcribed with miScriptII RT kit (Qiagen). miRNA expression was measured by real-time PCR using the miScript SYBR Green PCR kit (Qiagen). Quantification of miRNA expression was defined from the threshold cycle (Ct), and relative expression levels were calculated using the 2–ΔΔCt method after normalization to the SNORD95 expression level [38].

### Mass spectrometry (MS)

The cells transfected with flag tagged DAB2IP were lysed and immunoprecipitated with anti-flag antibody. The protein complex mixtures were run into the SDS-PAGE gel and stained with Coomassie blue. Identification of proteins in bands cut from Coomassie stained SDS-PAGE gel was performed by Orbitrap Elite mass-spectrometry platforms, using short reverse-phase LC-MS/MS method. Proteins were identified from samples using our in-house data analysis pipeline (CPFP) of UTSW proteomics core.

### Immunofluorescence (IMF) staining

Cells seeded on glass coverslips were fixed with 4% paraformaldehyde and permeabilized with 0.5% Triton X-100 for 20 min at room temperature. The samples were then blocked with 5% BSA for 1 h and incubated with first antibody overnight at 4^°^C. Samples were washed 3 times for 5 min in PBS, then incubated with secondary antibody for 1 h. Nuclei were counterstained with DAPI and stained cells were analyzed under a fluorescence microscope (Eclipse Ti, Nikon).

### Chromatin immunoprecipitation (ChIP) assay

ChIP assays were performed using ChIP-IT Express Enzymatic (Active motif) kit according to the manufacturer's instructions. Briefly, cells were fixed with 1% formaldehyde for 10 min and quenched with glycine. Nuclear extraction was isolated and chromatin was sheared into 200–100 bp using Enzymatic Shearing Cocktail. The sheared DNA was IP with DNMT1 as well as control IgG and then cross-links were reversed. The ChIP DNA was applied to quantitative real-time PCR.

### 3′-UTR reporter assays

Full-length 3′-UTRs of the miR-138-regulated targets ABCA13, EZH2 and Sox4 were amplified from human genomic DNA and individually cloned into psiCHECK-2 dual luciferase reporter vector (Promega). After transfection for 48 h, the ratio of *Renilla* to firefly luciferase was measured with the dual luciferase assay (Promega). All transfection and reporter assays were performed in triplicate.

### Sphere assay

Single cells were seeded in 96-well or 24-well ultralow attachment (ULA) plates (Corning, NY, USA) in serum-free prostate epithelial basal medium (PrEBM) supplemented with 4 μg/ml insulin, B27 (Life Technologies), and 20 ng/ml EGF and bFGF. Spheres that arose in 2 weeks were counted. To test the propagation of primary spheres, the spheres were collected by gentle centrifugation, then dissociated with trypsin-EDTA and mechanically disrupted with a pipette. The single cell was then centrifuged to remove the enzyme and re-suspended in media allowing reformation of spheres.

### Side population (SP) assay

After incubating with Hoechst 33342 (5 μg/mL) at 37^°^C for 90 min, the cells were washed with PBS and trypsinized. Collected cells were then filtered and resuspended in ice cold PBS and side population was analyzed by DakoCytomation MoFlo cytometer using dual-wavelength analysis (blue, 450/20 nm; red, 670 nm) after excitation with 350 nm UV light. Propidum iodie (5 μg/mL, Sigma) was added 5 minutes before analysis to define dead cells.

### Mouse xenografts

All animal work was approved by the Institutional Animal Care and Use Committee. Six weeks-old NOD SCID mice were orthotopically inoculated with varying number of cells. Briefly, cell suspension was mixed with Matrigel (BD Biosciences) 1:1 (v/v) to form a small sphere and approximately 50-μl volume was implanted beneath the kidney capsule. Tumor incidence was determined 6 weeks post injection and xenograft tissues were collected for the pathological examination.

### Statistical analysis

All error bars in graphical data represent mean ± SD. Student's two-tailed *t*-test was used for the determination of statistical relevance between groups, and *p* < 0.01 was considered statistically significant. All statistical analyses were performed with GraphPad Prism software.

## SUPPLEMENTARY MATERIALS FIGURES AND TABLE


