# Supplementary material for: The network of DAB2IP-miR-138 in regulating drug resistance of renal cell carcinoma associated with stem-like phenotypes
Source: Oncotarget. 2017 May 9;8(40):66975–86. doi: 10.18632/oncotarget.17756 (PMC5620150; doi:10.18632/oncotarget.17756)
Supplement: Supplementary file 1 [file oncotarget-08-66975-s001.pdf]

The network of DAB2IP-miR-138 in regulating drug resistance of renal cell carcinoma associated with stem-like phenotypes

SUPPLEMENTARY MATERIALS

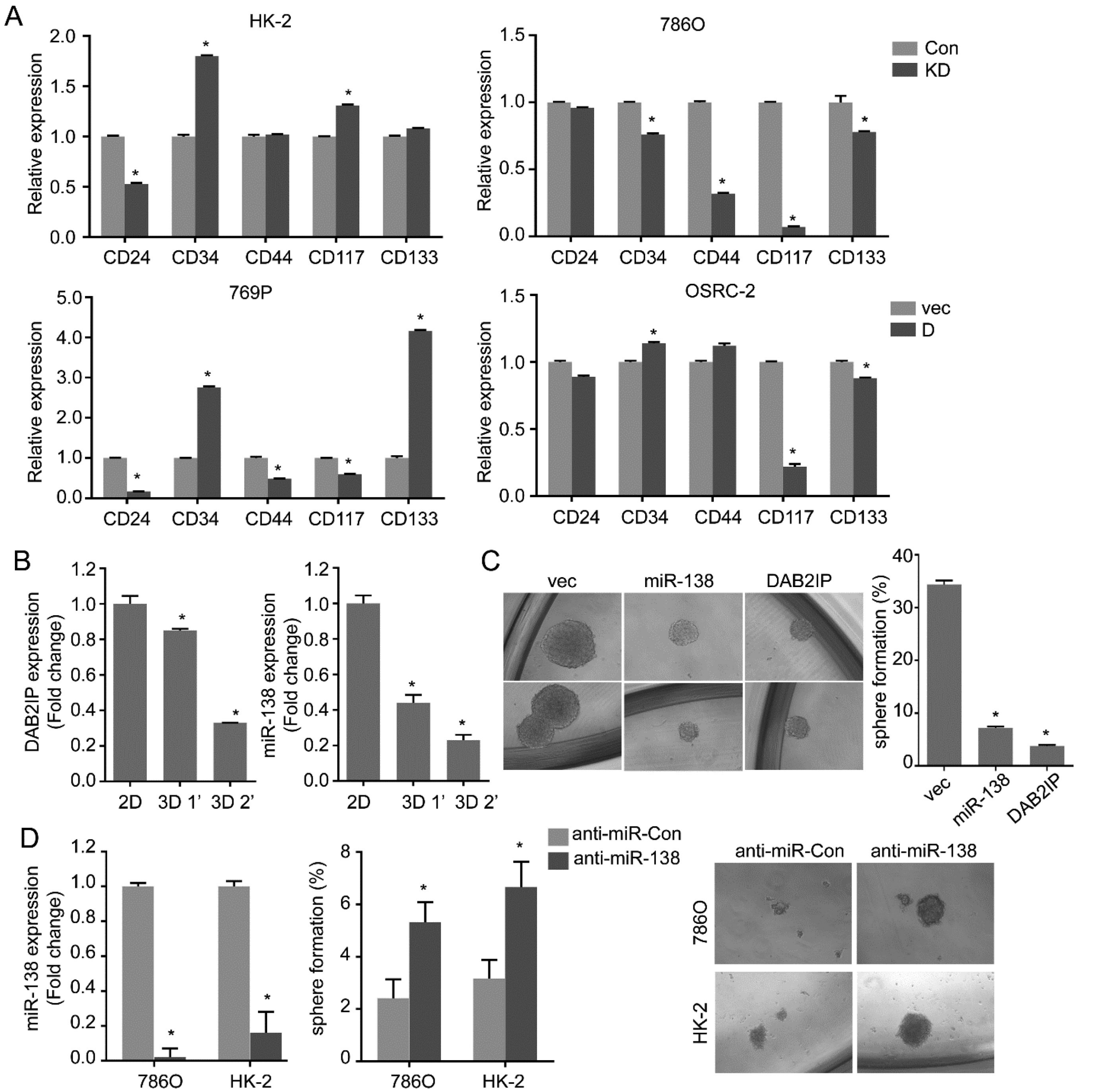

Supplementary Figure 1: The expression of DAB2IP and miR-138 inhibit stem-like cell properties of renal cells. (**A**) Expression levels of several stem cell markers by qRT-PCR. After normalizing with 18S rRNA in each sample, the relative mRNA levels were calculated using control (=1). (**B**) Profiling the expression of DAB2IP and miR-138 in HEK293T cells grown in monolayer (2D) or sphere condition (3D) were analyzed. (**C**) HK-2 KD cells were transfected miR-138 or DAB2IP expression plasmid, and the inhibitory effect of miR-138 and DAB2IP on sphere forming ability was determined. (**D**) 786O and HK-2 cells were transfected with anti-miR-138 plasmid then grown under ultra-low attachment condition for 2 weeks.

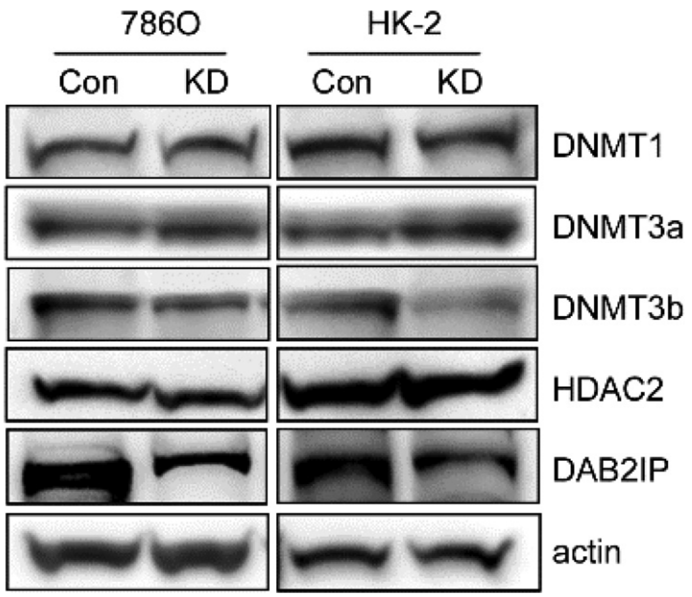

Supplementary Figure 2: DAB2IP does not regulate the expression of DNMT1 and DNMT3 protein. The expressions of DNMT1, DNMT3 and HDAC2 protein were compared in 786O and HK-2 sublines by Western blot analyses.

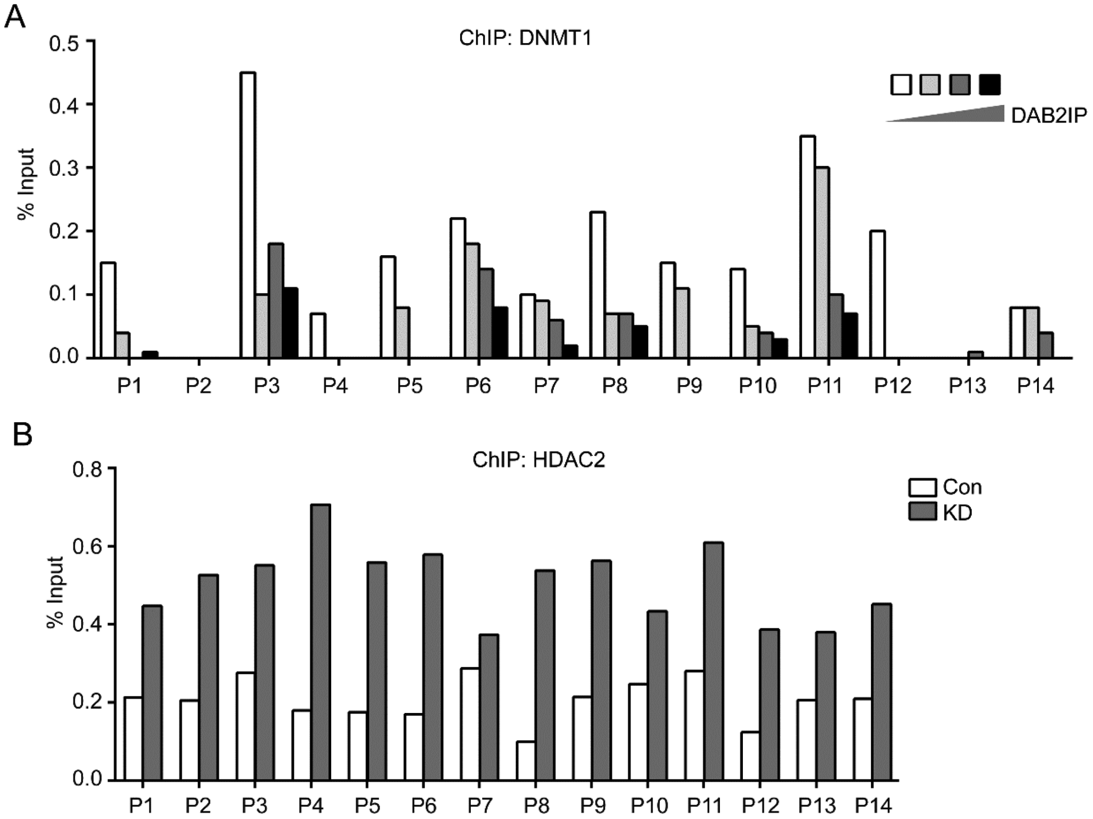

Supplementary Figure 3: DAB2IP inhibits the binding of DNMT1 to the miR-138 gene promoter. (**A**) 293 cells were transfected with different amount of DAB2IP plasmid and DNMT1 binding to the miR-138 gene promoter region was evaluated by ChIP assay. Sheared chromatin DNAs were immunoprecipitated with DNMT1 antibody and subjected to qRT-PCR. (**B**) The binding status of HDAC2 to the miR-138 gene promoter region in 786O Con and KD was evaluated by ChIP assay. (B) The binding status of HDAC2 binding to the miR-138 gene promoter region in 786O Con and KD was evaluated by ChIP assay.

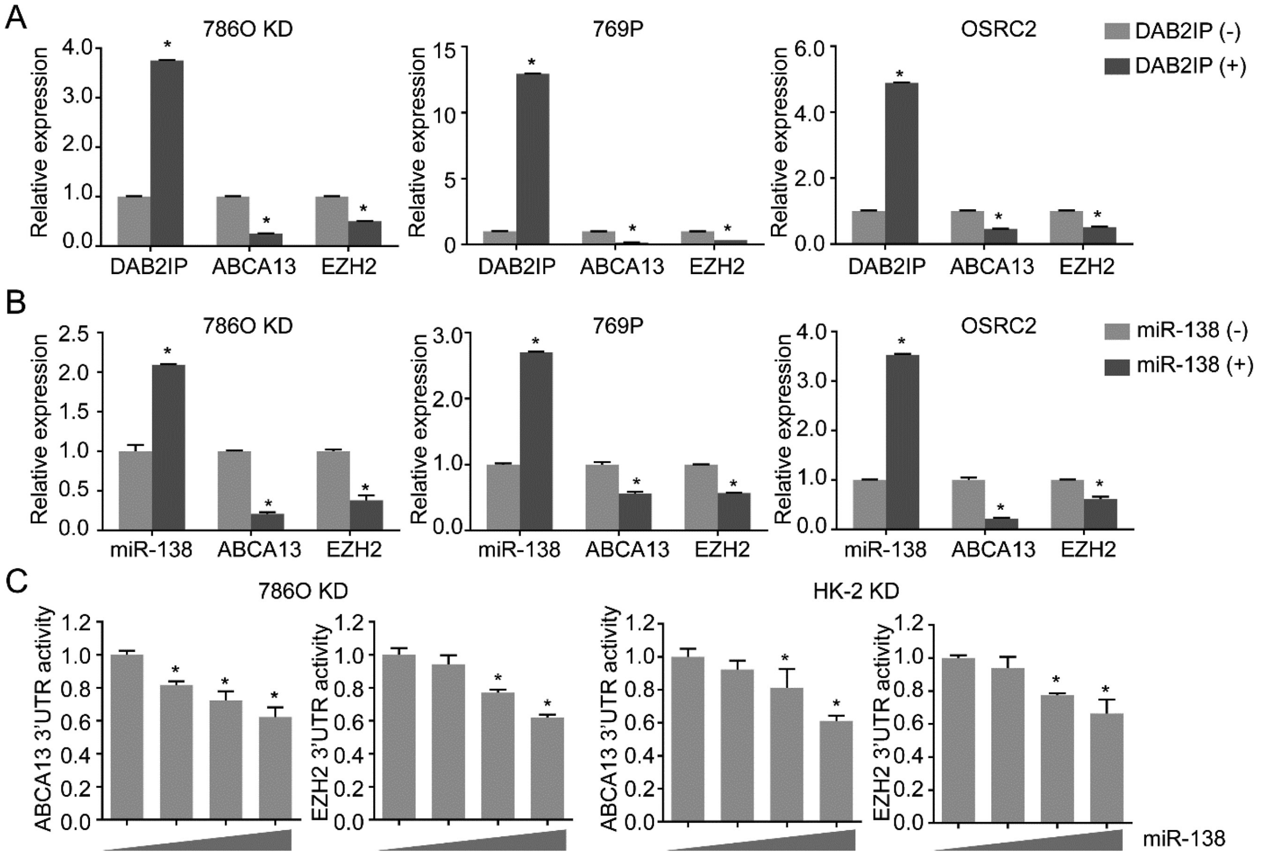

Supplementary Figure 4: miR-138 suppresses ABCA13 and EZH2 mRNA expression by targeting their 3′UTR region. (**A** and **B**) Cells were transiently transfected with DAB2IP (A) or miR-138 expression vector for 48 h and the expression levels of ABCA13 and EZH2 were analyzed by qRT-PCR. (**C**) Cells were co-transfected with incremental miR-138 expression vector and 3′UTR reporter construct for 48 h and luciferase reporter activities were determined.

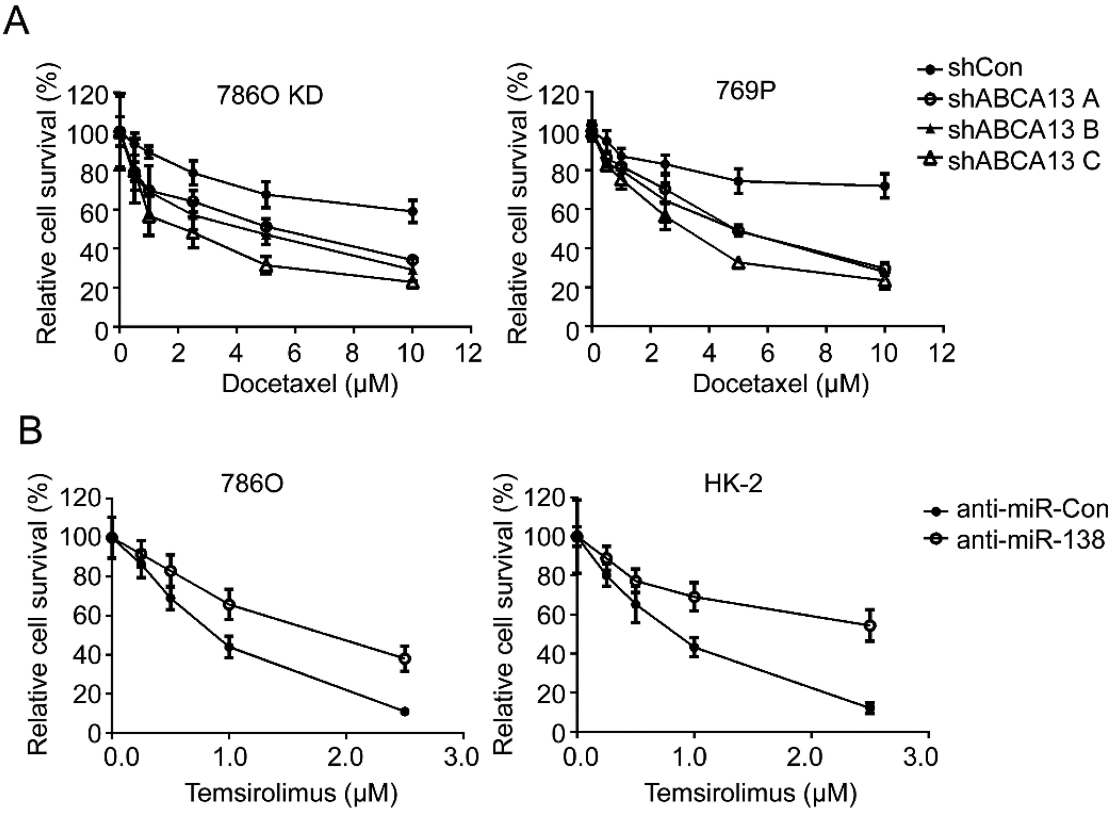

Supplementary Figure 5: Loss of miR-138 enhances drug resistance of RCC. (**A**) 786O KD or 769P cells were transfected with shCon or shABCA13 plasmid, then treated with Docetaxel for 48 h and the relative cell survival was measured by MTT assay for determination of IC_{50} using Graphpad Prism. (**B**) 786O or HK-2 cells transfected with anti-miR-Con and anti-miR-138 plasmid were treated with Temsirolimus for 72 h, and relative survival was measured by MTT assay.

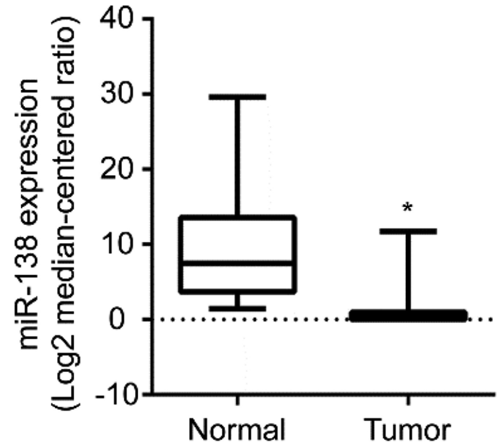

Supplementary Figure 6: Loss of miR-138 expression is associated with RCC specimens. The levels of miR-138 in normal versus tumor tissues from TCGA were analyzed.

Supplementary Table 1: The list of epigenetic modulators interacting with DAB2IP

Term	Gene List								
GO:0006304~ DNA modification	DNMT1	CTCF	HELLS						
GO:0006305~DNA alkylation	DNMT1	CTCF	HELLS						
GO:0006306~DNA methylation	DNMT1	CTCF	HELLS						
GO:0006342~chromatin silencing	HDAC2	HELLS	SMARCA4						
GO:0006730~one-carbon metabolic process	MTHFD1	KDM1A	GSPT1	DNMT1	CTCF	HELLS			
GO:0010216~maintenance of DNA methylation	DNMT1	CTCF	HELLS						
GO:0016441~posttranscriptional gene silencing	SRRT	SND1	CELF1						
GO:0016458~gene silencing	SRRT	HDAC2	SND1	CELF1	HELLS	SMARCA4	ADAR		
GO:0016569~covalent chromatin modification	KDM1A	HDAC2	SMARCA4	HELLS	BAZ1B				
GO:0031047~gene silencing by RNA	SRRT	SND1	CELF1	ADAR					
GO:0032259~methylation	GSPT1	DNMT1	CTCF	HELLS					
GO:0035194~posttranscriptional gene silencing by RNA	SRRT	SND1	CELF1						
GO:0040029~regulation of gene expression epigenetic	SRRT	HDAC2	SMARCA4	DNMT1	SND1	CELF1	HELLS	CTCF	
GO:0043414~biopolymer methylation	GSPT1	DNMT1	CTCF	HELLS					
GO:0045814~negative regulation of gene expression epigenetic	HDAC2	HELLS	SMARCA4						
GO:0000118~histone deacetylase complex	SATB2	HDAC2	MTA2	CHD4					
GO:0000792~heterochromatin	TCP1	HDAC2	SMARCA4	SMARCC1	BAZ1B	TOP2B	HELLS	TRIM28	
GO:0016581~NuRD complex	HDAC2	MTA2	CHD4						
GO:0016585~chromatin remodeling complex	SATB2	HDAC2	SMARCC1	SMARCD2	BAZ1B	SMARCC2	MTA2	SMARCA5	CHD4
GO:0017053~transcriptional repressor complex	HDAC2	MTA2	SMARCC2	CHD4					
